# Application of Plant Antimicrobials in the Food Sector: Where Do We Stand?

**DOI:** 10.3390/foods13142222

**Published:** 2024-07-15

**Authors:** Loris Pinto, Jesús Fernando Ayala-Zavala

**Affiliations:** 1 Institute of Sciences of Food Production, National Research Council of Italy, Via G. Amendola 122/O, 70126 Bari, Italy; 2 Centro de Investigación en Alimentación y Desarrollo, A.C., Carretera Gustavo Enrique Astiazarán Rosas 46, Hermosillo 83304, Sonora, Mexico; jayala@ciad.mx

## Abstract

The Special Issue “Plant Extracts Used to Control Microbial Growth: Efficacy, Stability and Safety Issues for Food Applications” explored the potential of plant-based extracts as natural antimicrobial agents in the food industry. Its purpose was to address the growing demand for natural, safe, and effective food preservation methods. The contributions highlighted various plant extracts’ antimicrobial efficacy, including phenolic compounds, terpenes, and other bioactive substances. Research papers and one review were submitted from countries, including Spain, Portugal, Italy, Mexico, Turkey, India, USA, Romania, China, and Lithuania, showcasing a diverse international collaboration. Key topics covered in this issue included the chemical characterization of plant extracts, their stability under different processing and storage conditions, and their safety assessments. Advances were reported in using plant extracts to inhibit spoilage microorganisms and foodborne pathogens, enhance food safety, and extend shelf life. The published papers in the Special Issue studied various food types, including yogurt, catfish fillets, edible Mushrooms, red grapes, herring Fillets, and various food types covered in the review. This diversity demonstrates the broad applicability of plant extracts across different food products. Notable findings included the antimicrobial activities of fermented grapevine leaves, grapefruit seed extract, cinnamaldehyde, clove oil, and other plant-based compounds. In conclusion, this Special Issue demonstrated significant progress in applying plant extracts for food preservation, highlighting their potential to contribute to safer and more sustainable food systems worldwide.

## 1. Utilizing the Power of Plant Antimicrobials for Food Safety

The use of natural preservatives with antimicrobial properties has gotten significant interest in food and drug research due to the growing awareness of the negative impacts associated with synthetic preservatives. These impacts include potential health risks to consumers, the emergence of multidrug-resistant microorganisms, and the requirement for alternatives to traditional thermal treatments [1,2,3]. Plant antimicrobials offer a promising solution as they are rich sources of multiple bioactive compounds capable of reducing contamination levels of pathogenic bacteria and inhibiting the growth of spoilage microorganisms in various foods. The compounds found in plant antimicrobials are diverse and include polyphenols known for their antioxidant properties; polyphenols also exhibit strong antimicrobial activity [4,5]. They can disrupt microbial cell membranes and interfere with the functions of microbial enzymes and proteins [6,7]. Essential oils and their constituents, such as carvacrol, thymol, and eugenol, possess great antimicrobial properties [8,9,10]. Essential oils can penetrate microbial cell membranes, causing structural and functional damage [11,12]. Glucosinolate derivatives found in cruciferous vegetables have been shown to possess antimicrobial activity against a broad spectrum of microorganisms [13]. They act by releasing isothiocyanates upon hydrolysis, which are toxic to bacteria [14]. Alkaloids are nitrogen-containing compounds with antimicrobial properties that can inhibit the growth of bacteria, fungi, and viruses [15]. Alkaloids can interfere with DNA replication and protein synthesis in microorganisms and can attenuate bacterial pathogenesis [15,16]. Thiols are sulfur-containing compounds that exhibit strong antimicrobial activity [17]. They can disrupt microbial cell walls and membranes, leading to cell lysis and death.

The objective of the Special Issue entitled “Plant Extracts Used to Control Microbial Growth: Efficacy, Stability and Safety Issues for Food Applications” was to present the latest advances in the use of plant antimicrobials to reduce spoilage microorganisms and ensure food safety across different foodstuffs. The papers in this issue highlight the potential of plant extracts as natural preservatives that can be integrated into various food systems to enhance safety and extend shelf life.

## 2. Summary of Published Papers

The main studied sources of plant antimicrobials were the herbal plant *Potentilla kleiniana*, cranberry pomace and grape seeds, essential oils and their compounds, bog bilberry leaf extracts, spice extracts, grapefruit seed extract, and grapevine leaf extracts (Table 1). Among the compounds in the used raw extracts were identified phenolic acids, flavonoids, and terpenes from plant tissues.

Tang et al. evaluated the antibacterial activity of the methanol phase extract from the edible herb *Potentilla kleiniana* against more than 20 pathogenic bacteria. MIC values of Fragment 1 ranged from 6.25 to 50 mg/mL against *Bacillus cereus*, *Shigella flexneri*, *Staphylococcus aureus*, and *Vibrio parahemolyticus* strains. Oxymorphone and rutin were identified in Fragment 1, and a putative mechanism of action involving the inhibition of energy supply and protein translation, the blocking of signal transduction, and the repression of ABC transporters was proposed [Contribution 1]. An alginate/pectin film containing grape seed extract showed bacteriostatic activity against *L. monocytogenes* on herring, reducing its load by 3 log cfu/g compared to unpacked fillets stored for 18 days at 4 °C. In addition, the accumulation of histamine, cadaverine, putrescine, and tyramine was significantly reduced starting from 12 days of storage in fillets packed using the active coating [Contribution 2].

Li et al. found that carvacrol displayed the lowest EC50 value against *A. alternata*, significantly reducing the decay rate when applied to contaminated red grapes. Grapes inoculated with *A. alternata* showed a decay rate higher than 60%, whereas contaminated fruit treated with carvacrol showed a decay rate lower than 15% after 12 days at room temperature [Contribution 3]. Bog bilberry leaf extracts obtained through ultrasound (UAE) extraction showed the lowest MIC values against *Candida parapsilosis* and *Salmonella enterica*; high-pressure (HPE) extracts showed the inhibition of *S. aureus* growth at sub-MIC levels [Contribution 4]. Kuley et al. demonstrated that, among four spice extracts, sumac (*Rhus coriaria* L.) extract reduced by 1–3 log cfu/mL the growth of *Enterococcus faecalis*, *Campylobacter jejuni*, and *Yersinia enterocolitica* in tyrosine decarboxylase broth and the production of histamine by *E. faecalis* and tyramine by *C. jejuni* [Contribution 5].

Grapefruit seed extract showed antibacterial action against *S. aureus* ATCC 6538, *P. aeruginosa* ATCC 9027, *P. fluorescens* wild type, *Escherichia coli* ATCC 8739, with MIC values ranging from 162.5 µg/mL to 650 µg/mL. Rutin, naringin, hesperidin, neohesperidin, and naringenin were identified in the extract. A preliminary trial on mushrooms showed that applying grapefruit seed extract can limit the development of yellowing on *Pleurotus eryngii* caused by *Pseudomonas* spp. [Contribution 6]. Cinnamaldehyde and clove oil showed in vitro antibacterial activity against *S. baltica* and *A. hydrophila*; their application on adsorbent pads in contact with catfish fillets reduced the total bacteria on pads by 3 to 6 log cfu/mL [Contribution 7].

Freitas et al. found that fermented grapevine leaves using *Saccharomyces cerevisiae*, in both solid and liquid media, preserved the microbiological quality of yogurt in the same manner as potassium sorbate without affecting the viability of lactic acid bacteria. Further research is necessary to evaluate the antimicrobial effect of fermented grapevine leaves against yogurt spoilage microorganisms [Contribution 8]. Finally, Pinto et al. summarized recent findings on applying plant extracts and plant antimicrobial compounds against spoilage and pathogenic microorganisms in different foods. Interesting results were achieved by using combinations of plant antimicrobials, with synergistic or additive effects, and by integrating plant extracts with food technologies, ensuring an improved hurdle effect. The review highlighted the need for further research in fields such as the mode of action of plant antimicrobials, optimization of delivery systems, sensory properties of food including plant antimicrobial compounds, safety assessment of plant extracts, regulatory aspects, eco-friendly production methods, and consumer education [18].

## 3. Key Advances and Findings

The papers published in this issue highlighted several key advances and findings, demonstrating the potential of plant-based compounds to enhance food safety and quality. The methanol-phase extract from *Potentilla kleiniana* exhibited a 68% inhibition against over 20 pathogenic bacteria, with MIC values ranging from 1.56 to 50 mg/mL. This study identified oxymorphone and rutin as active components and proposed mechanisms involving inhibiting energy supply, protein translation, signal transduction, and repression of ABC transporters. Alginate/pectin films containing cranberry pomace and grape seed extracts showed bacteriostatic activity against *Listeria monocytogenes* on herring, significantly reducing the load of histamine and cadaverine during storage. Carvacrol was effective against *Alternaria alternata* in red grapes, reducing the decay rate to less than 15% compared to over 60% in untreated controls after 12 days at 25 °C. Bog bilberry leaf extracts obtained through ultrasound extraction showed the lowest MIC values against *Candida parapsilosis* and *Salmonella enterica*, with high-pressure extracts inhibiting *Staphylococcus aureus* at sub-MIC levels. Sumac extract significantly reduced the growth of several foodborne pathogens, including *Enterococcus faecalis*, *Campylobacter jejuni*, and *Yersinia enterocolitica*, and decreased the production of biogenic amines.

Grapefruit Seed Extract demonstrated broad-spectrum antibacterial activity against multiple strains, including *S. aureus, P. aeruginosa, P. fluorescens*, and *E. coli*, with MIC values between 162.5 µg/mL and 650 µg/mL. Additionally, it showed potential in preventing yellowing in mushrooms caused by *Pseudomonas* spp. Cinnamaldehyde and clove oil applied on absorbent pads in contact with catfish fillets reduced total bacterial counts by 3 to 6 log cfu/mL. Fermented grapevine leaves used in yogurt maintained microbial quality similar to potassium sorbate without compromising the viability of lactic acid bacteria.

Several studies focused on improving the stability of plant extracts under different processing and storage conditions. Techniques such as encapsulation [19,20,21], inclusion in biopolymers [22,23,24], and spray-drying [25,26,27] were explored to enhance the stability and effectiveness of plant antimicrobials. The research covered a wide range of food products, demonstrating the versatility of plant antimicrobials. These included yogurt, herring fillets, red grapes, catfish fillets, and mushrooms. This diversity illustrates the broad applicability of plant extracts across different food matrices, contributing to enhanced food safety and shelf life. Combining different plant extracts often resulted in synergistic or additive antimicrobial effects. This approach and integration into food technologies provided an improved hurdle effect, enhancing overall food preservation outcomes.

## 4. Future Research Directions

The Special Issue highlighted the need for further research in several key areas. Detailed studies are necessary to understand how plant antimicrobials exert their effects, focusing on their mode of action. Additionally, there is a need for the optimization of delivery systems to develop effective methods that maximize the efficacy of plant extracts. Investigating the impact of plant antimicrobials on the sensory attributes of food is crucial to ensure that these natural preservatives do not negatively affect taste, texture, or aroma. Comprehensive safety evaluations are required to ensure consumer health is not compromised. Addressing regulatory challenges will facilitate the commercial use of plant antimicrobials, ensuring they meet all necessary standards and guidelines. Developing sustainable production techniques for plant extracts is essential to promote eco-friendly methods that align with environmental goals. Lastly, increasing awareness and acceptance of natural food preservatives among consumers through effective education campaigns is vital for broader adoption and understanding of these innovations.

## 5. Conclusions

This Special Issue has significantly contributed to the field of food microbiology by advancing our knowledge of plant antimicrobials and their applications. The findings emphasize the potential of these natural compounds to revolutionize food preservation, paving the way for safer, more sustainable food systems that meet the growing demand for natural and health-friendly food additives.

## Figures and Tables

**Table 1:** Plant antimicrobial compounds, sources, target food/microorganisms, and main results were obtained in the published papers in this special issue.

Plant Antimicrobial Compounds	Source	Target Food/Microorganisms	Main Results	Contribution
Oxymorphone and rutin	Methanol-phase extract from an edible herb *Potentilla kleiniana* Wight et Arn	More than 20 pathogenic bacteria	Inhibition rate of 68%, MIC values of 1.56–50 mg mL^−1^, putative mechanism of action	[1]
Polyphenols and procyanidins	Cranberry pomace and grape seed extracts	Herring/*Listeria monocytogenes* and *Pseudomonas aeruginosa*	Film with grape seed extract showed bacteriostatic activity against *L. monocytogenes* and reduced the concentration of histamine and cadaverine	[2]
Carvacrol, thymol, geraniol, citral, L-menthol, menthone, anisaldehyde, linalool, citronellal, trans-2-hexenal, diallyl disulfide, trans-caryophyllene, piperone, eugenol, and anethole	RON Reagent Shanghai Yi En Chemical Technology Co., Ltd., Shanghai, China	Red grape fruit/*Alternaria alternata*	Significant reduction of the decay rate after carvacrol treatment	[3]
Phenolic acids, flavonols, flavanols	Leaf extracts of the bog bilberry	Gram-positive and Gram-negative bacteria, yeasts	MIC values of 8.9 or 17.8 mg mL^−1^	[4]
Polyphenolic compounds	Sumac (*Rhus coriaria* L.), cumin (*Cuminum cyminum* L.), black pepper (*Piper nigrum)*, and red pepper (*Capsicum annuum*) extracts	Gram-positive and Gram-negative bacteria	Sumac extract reduced the growth of foodborne pathogens and the production of biogenic amines	[5]
Flavonoids	Grapefruit seed extracts	Mushroom/Gram-positive and Gram-negative bacteria, yeast	MIC values from 162.5 µg mL^−1^ to 650 µg mL^−1^, potential application to reduce yellowing on mushrooms	[6]
Cinnamaldheydhe and clove oil	Sigma-Aldrich (St. Louis, MO, USA) and Piping Rock Health Products LLC (Ronkonkoma, NY, USA)	Catfish fillet/*Shewanella baltica*, *Aeromonas hydrophila,* total bacteria	Reduction of 3 or 6 log cfu mL^−1^ of total bacteria on adsorbent pads	[7]
Polyphenols	Fermented grapevine leaves	Total yeasts and bacteria of yogurt	Fermented grapevine leaves showed a preserving effect equal to potassium sorbate	[8]
